# Is Leukotriene Receptor Antagonist the Direct Cause of Churg-Strauss Syndrome in Asthmatic Patients?

**DOI:** 10.7759/cureus.28018

**Published:** 2022-08-14

**Authors:** Salomi Paul, Shreyas Yakkali, Sneha Teresa Selvin, Sonu Thomas, Viktoriya Bikeyeva, Ahmed Abdullah, Aleksandra Radivojevic, Anas A Abu Jad, Anvesh Ravanavena, Chetna Ravindra, Emmanuelar O Igweonu-Nwakile, Safina Ali, Pousette Hamid

**Affiliations:** 1 Medicine, California Institute of Behavioral Neurosciences & Psychology, Fairfield, USA; 2 Internal Medicine, California Institute of Behavioral Neurosciences & Psychology, Fairfield, USA; 3 Behavioral Neurosciences and Psychology, California Institute of Behavioral Neurosciences & Psychology, Fairfield, USA; 4 General Surgery, California Institute of Behavioral Neurosciences & Psychology, Fairfield, USA; 5 Neurology, California Institute of Behavioral Neurosciences & Psychology, Fairfield, USA

**Keywords:** eosinophilic infiltrates, anca associated vasculitis, vasculitis, inhaled corticosteroids, steroid tapering, asthma, leukotriene receptor antagonists, churg strauss syndrome

## Abstract

One of the main reasons for continuous, persistent asthma is when there is a change in the structure of the airways and the Lung parenchyma. These persistent changes bring a much worse prognosis to asthmatic conditions and predispose the situation to severe asthmatic syndromes such as Churg-Strauss syndrome (CSS). CSS is an inflammation of systemic blood vessels and is a rare disorder that can be suspected in long-standing asthmatic patients. Leukotriene antagonists receptor antagonists (LTRA) have been used to treat asthma along with tapering steroids. But after the introduction of LTRA therapy in these patients suggests a causal relation between LTRA initiation and the development of CSS, or it is an unmasking of CSS as the dose of steroid tapers down with LTRA therapy. This review highlights the relationship between leukotriene antagonists and the pathogenesis of CSS. It summarizes the current literature regarding the development of CSS with the initiation of LTRA therapy on asthmatic patients. The literature on this topic was reviewed using different research/article searches, manual library searches, conference abstracts, and internet searches.

## Introduction and background

Churg-Strauss syndrome (CSS) is a disorder marked by blood vessel inflammation. CSS is a rare syndrome associated with asthma and eosinophilia in tissues and peripherals. There are three phases related to CSS-prodromal, eosinophilic, and vasculitis, and they do not always occur successively [1,2]. Leukotriene receptor antagonists (LTRA) have been introduced as the new mode of therapy for asthma. LTRA has been in the market for the last 10 years, and they play an essential role in treating Asthma by sparing the steroid in the inflammatory Component of Asthma. Following the introduction of LTRA to asthmatic patients, there have been several reports of CSS and realized this as a possible side effect in individuals suffering from moderate to severe asthma and on LTRA therapy.

**Figure 1:**
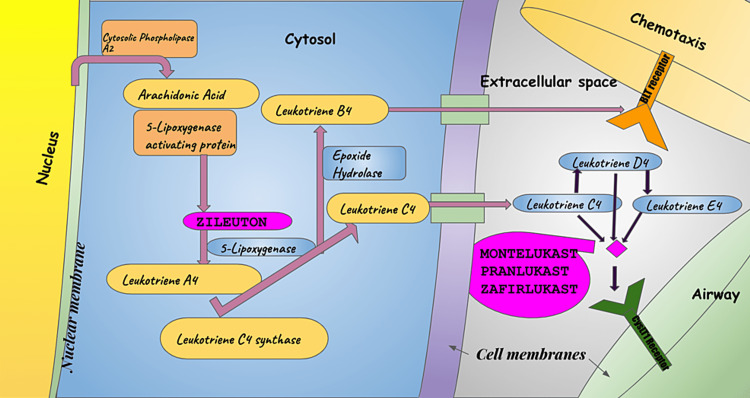
Physiological effects and biosynthesis of leukotrienes and the pharmacological actions of antileukotriene. Adapted from [7]

Leukotrienes are produced by several cells involved in the asthmatic response, including eosinophils, mast cells, monocytes, and macrophages, and they are found in bronchoalveolar lavage fluid asthmatics [3]. The effects of leukotrienes include bronchoconstriction, mucus secretion, vascular permeability, decreased mucociliary clearance, edema, and eosinophil recruitment to the airways-these effects build up to chronic inflammation, which contributes to airway remodeling [4,5]. The Discovery of Leukotrienes began over 60 years ago, and they were initially termed as a “slow-reacting substance of anaphylaxis” (SRS-A) - quoted by Brocklehurst in 1960 [6]. The physiological effects and Biosynthesis of Leukotrienes and the pharmacological actions of leukotriene antagonists are shown in Figure 1.

The American College of Rheumatology (ACR) proposed six criteria for diagnosing CSS [8]. Out of these six criteria, four were needed for CSS to be diagnosed with 99.7% specificity and 85% sensitivity. Some reports suggest males being affected more than females by CSS. The age at risk for CSS is between 30 and 50 years, even though the disorder can develop in people of almost any age, from 15 to 70 years of age. The estimated mean annual incidence is 2.4 people per million. But according to Kurland. Lt and the team, the incidence of CSS is one-two cases per million persons per year in the general population: this may range from 0.3 to 4 per million persons per year [9]. And for other few researchers, CSS is an under-diagnosed disease, making it difficult to evaluate its actual frequency among the population.

Though inhaled corticosteroids and bronchodilators work in most patients with asthma, numerous individuals respond poorly to these medications. They frequently require intermittent courses of corticosteroids via oral or, at times, they depend on long-term oral corticosteroid treatment. LTRA has succeeded in reducing the need for steroids in patients with difficult asthmatic situations but also seems to be unmasking some undiagnosed cases of CSS, the symptoms of which were being controlled by steroids. There has been some concern that LTRA might precipitate the onset of CSS. Different studies were conducted to understand the relationship between LTRA and CSS. Some claim that severe neuropathy can be the first clinical feature of CSS with the long-standing treatment with LTRA [10]. However, the genuine relationship between LTRA and CSS is still not known. This study aims to bring more understanding and rule out the unnecessary causes of CSS if any.

## Review

The CSS was first described in 1951 by two physicians, Jacob Churg and Lotte Strauss, hence named Churg-Strauss syndrome. Jacob Churg and Lotte Strauss reported a case series of 13 patients based on autopsy findings. The 13 patients mentioned in the case series were dealing with severe asthma and were later diagnosed with granulomatosis (allergic) and angiitis. They were looking for three features to define their diagnosis [11,12] and those were - eosinophilic infiltration, necrotizing inflammation of small and medium-sized vessels, and formation of extravascular granuloma.

The common findings of their histology were necrotizing vasculitis, granuloma, which is necrotizing and centered on necrotic eosinophils, tissue, and blood eosinophilia. There were patients with other signs and symptoms, which made the criteria problematic in diagnosing CSS. Hence, there came a need to develop new clinically relevant diagnostic criteria. 

Then, corticosteroid therapy became the preferred treatment, and among the patients who survived this therapy were only two. Thirty years later, Lanham et al. reviewed 154 cases diagnosed with CSS [13]. After studying and understanding, they found out that corticosteroids could be successfully used for CSS. They also came up with different diagnostic criteria and phasic patterns (Table 1). Tissue eosinophilia was considered the early stage of the CSS, whereas the life-threatening stage of CSS is a vasculitic phase. Apart from the above criteria, the ACR also came up with six criteria in 1990, where four are essential to diagnose CSS. Also, anti-neutrophil cytoplasmic antibodies (ANCA) became available, and it was found that 60%-70% of people suffering from CSS had positive ANCA, which became a confirmatory test for CSS later [14].

**Table 1:** Different phases of Churg-Strauss syndrome

PHASE-1	PHASE-2	PHASE-3
Prodromal phase	Eosinophilic Phase	Vasculitic Phase
Late-onset Allergic Disease	Peripheral eosinophilia	Constitutional symptoms
Evidence of Asthma	Eosinophilic inflammation in the tissue	End Organ vasculitis
Sinusitis	Pulmonary infiltrates	Mononeuritis multiplex
Myalgias, arthralgias	Vasculitis(Necrotizing), granulomas	Subcutaneous skin nodules
Blood eosinophilia	Eosinophilic gastroenteritis	Kidney disease

After introducing LTRA in the management of severe Asthma, the link between the LTRA and CSS has been reported. After the initiation of the LTRA, most of the patients were found to have CSS in three days to nine months. It was also found that Zafirlukast is the most reported anti-leukotriene associated with CSS [15,16]. Another Leukotriene receptor blocker, Montelukast, became available on the market in 1998. Though Zafirlukast's chemical structure differs from Montelukast, several cases related to CSS have been reported with Montelukast and Pranlukast [17]. Even though LTRA became the first-class drug for asthma in the last 30 years [18], there is always conflicting evidence on whether the association between LTRA and CSS is primarily a result of confounding by inkling or is a genuine causal link. For example, two papers, one published in this issue of Thorax, and the other published recently, utilizing different methodologies, have addressed this issue and came to different conclusions.

Nathani et al. [19] presented a systematic review of cases and case series that report CSS and LTRA use. They identified 63 cases who developed CSS after LTRA therapy. The mean age at presentation was 47 (range 7-79) years, and 33 (55%) were female. Before the presentation, the mean duration of asthma was 7.5 years (range from four months to 20 years) [19]. One among the 63 cases was excluded as it could not meet The ACR criteria. The other 62 instances met at least four out of six ACR criteria. The patients that have been reported did not have variations in the diagnostic criteria. They conducted this study by dividing patients into three groups as shown in Table 2.

**Table 2:** Categories that define each group for the study conducted by Nathani and her team

Groups	Studies
GROUP 1	Received no previous steroid therapy and was introduced to LTRA therapy.
GROUP 2	Already introduced to inhaled or oral steroid therapy but with no change in the steroids after introducing LTRA
GROUP 3	Clear reduction in the steroid therapy after LTRA has been introduced.

**Figure 2:**
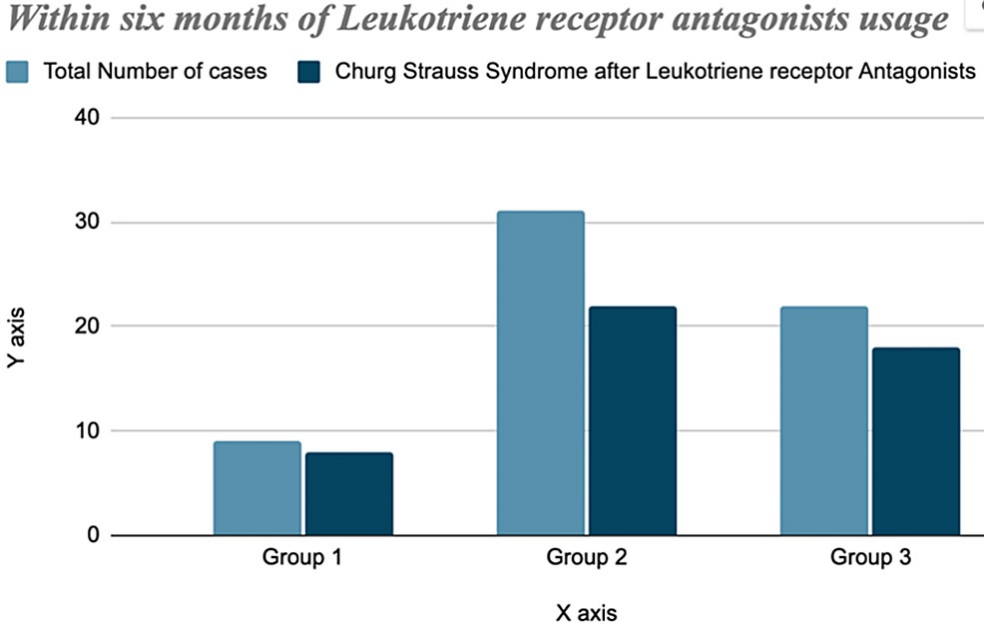
Three groups with Churg-Strauss syndrome after leukotriene receptor antagonists vs total number of cases in each group within six months.

During their studies, they found out that after the introduction of LTRA therapy, CSS was reported post 12 months in only five out of 62 patients, that is 8.5%. Rest of the cases were reported within the six months of initiation of LTRA therapy. That is almost eight out of nine cases in the first group (89%), 22 out of 31 (71%) in the second group) and 18 out of 22 cases (83%) in the third group presented with CSS within six months of initiation of LTRA, as shown in Figure 2 [19].

**Figure 3:**
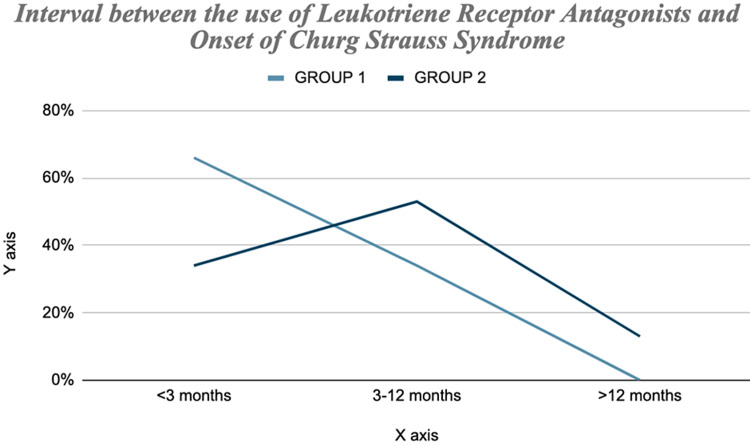
The percentages refer to the fraction of each group developing Churg-Strauss Syndrome in the time scale specified.

**Figure 4:**
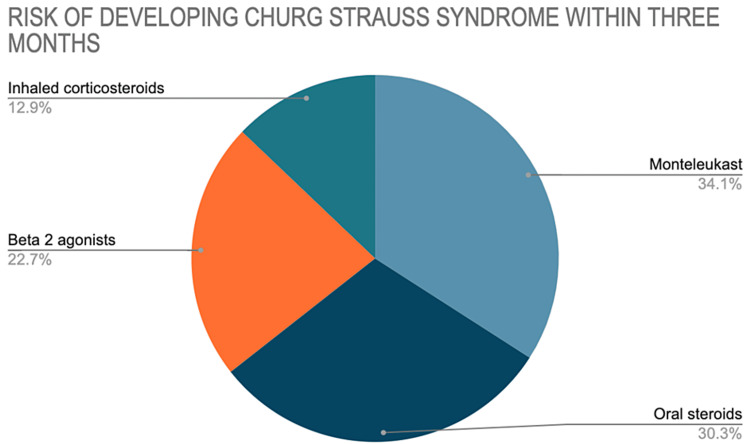
Pie chart shows the multivariable analyses of the Hausen and team and their results within three months.

In a different publication, Hauser et al. [20,21] investigated the exposure to the LTRA Montelukast (and other asthma medications) and the progression of CSS by an extension of the case-control methodology. They studied with two databases by doing a Case-Control study. One database was a French Cohort, and the other a German Cohort. They identified 78 patients with these two databases, and these groups were part of a prospective registry for a tertiary referral center for vasculitis management. They accumulated the databases over 10 years, and their study focused completely on Montelukast because Montelukast was the only LTRA approved in Germany and France. During this study, they found out that the use of Montelukast in asthmatic patients within three months before the development of CSS was more likely than use in the period more than three months before its development as shown in Figure 3. That optimistic estimates were also obtained for other long-term asthma control medications with effects independent of Montelukast use. During the first three months of the treatment, Montelukast showed 4.5 fold higher risk for CSS development as shown in Figure 4. Hence, based on their 10-year study and the collected data, the authors concluded that the CSS connection is specific to Montelukast and it also developed with other asthmatic therapies. It was more likely to be caused by a general treatment escalation in response to worsening Asthma. The authors also noted that there was increased use of Montelukast during their study period, adding weight to a confounding rather than a causative role of Montelukast in CSS development. They also found out that a case-crossover design qualified for a better assessment of the association between LTRA and CSS, compared to the traditional case-control study because it removes the daunting task of selecting a reasonable control group.

The conclusion of both papers highlights the fact that CSS is rare and so it is not easy to detect significant associations in statistical testing. Both studies are also likely to be influenced by selection bias [21], which may limit the generalizability of the findings. However, the systematic review by Nathani and colleagues does provide important information that CSS may occur in association with LTRA therapy in the absence of pre-existing disease, may appear on rechallenge with LTRA therapy [19,21,22], and may remit on the withdrawal of LTRA therapy without the need to start or increase the dose of systemic steroid or immunosuppressive therapy [21]. In the case of Hauser and colleagues, the details they provided in the case-control study are open to conflict even though the authors could identify a strong interconnection between CSS and LTRA therapy but could not convince their demonstrations regarding the clue that led to this relation.

The increase in risk with oral steroids is likely to relate to their use in severe exacerbations of sthma resulting from the progression of CSS [21]. Notably, Hauser and colleagues have provided ample evidence (consistent with the previous case-control study) that it is likely to have been selection bias in the association between asthma therapy and CSS. The vast majority of reported cases are reported to relate to LTRA therapy. In contrast, such cases represented only a quarter of their database.

As per Sloans et al.'s studies, leukotriene antagonists act as a causative role in CSS development, and other authors defend this suggestion through their investigations [23]. All the cases that have been reported are suggestive of the transparent temporal relationship between the progression of CSS and the use of these drugs. Moreover, the CSS had advanced not only with Montelukast but also with Pranlukast and Zafirlukast. This suggests that CSS may be linked to the effect of LTRA drugs on Leukotriene receptors. The annual increase in the incidence of the CSS has been reported ever since leukotriene antagonist drugs were introduced among asthmatic patients. Sloan and the team recollected that three out of four patients diagnosed with CSS in their department in a single year were related to using LTRA. During the last 20 years in their hospital, a total of 32 patients have been diagnosed and showed an increase in their yearly incidence with one to two new cases of CSS per year [23-26].

## Conclusions

These studies conclude that the data collected from different scenarios suggest a close relation between leukotriene antagonists and CSS development in asthmatic patients. This connection is found to be a causal link in some of the studies. According to Hauser et al., Montelukast was found to hold 4.5 times higher risk of developing CSS in three months. They claim that the Montelukast is a confounding rather than a causative role in the development of CSS. Nathani et al. remind clinicians, especially respiratory physicians, to watch out for the risk of CSS after introducing LTRA therapy to asthmatic patients. They also remind the clinicians to make patients look after the symptoms and signs of CSS after taking LTRA therapy. They say that at least two criteria of The ACR should allow the physician to explore the possibility of vasculitis before the LTRA therapy introduction. Also, Sloan et al. explain that there has been an increase in CSS incidence after the Leukotriene modifiers introduction. Based on the studies, the clinicians/healthcare providers should monitor the patients throughout the LTRA therapy, particularly with moderate to severe asthma or in the setting of steroid withdrawal. Based on this review and all the data collected from different studies, it is clear that LTRA therapy has a higher chance of being a causal link to the CSS in asthmatic patients.
